# The role of miRNAs in the invasion and metastasis of cervical cancer

**DOI:** 10.1042/BSR20181377

**Published:** 2019-03-15

**Authors:** Jin-yan Wang, Li-juan Chen

**Affiliations:** 1 Department of Obstetrics and Gynecology, Zhangjiagang First People’s Hospital, Zhangjiagang 215600, Jiangsu, P.R. China; 2 Department of Oncology, The First Affiliated Hospital of Nanjing Medical University, Nanjing 210029, P.R. China

**Keywords:** cervical cancer, invasion, MicroRNAs, metastasis

## Abstract

Cervical cancer (CC) with early metastasis of the primary tumor results in poor prognosis and poor therapeutic outcomes. MicroRNAs (miRNAs) are small, noncoding RNA molecules that play a substantial role in regulating gene expression post-transcriptionally and influence the development and progression of tumors. Numerous studies have discovered that miRNAs play significant roles in the invasion and metastasis of CC by affecting specific pathways, including Notch, Wnt/β-catenin, and phosphoinositide-3 kinase (PI3K)-Akt pathways. miRNAs also effectively modulate the process of epithelial–mesenchymal transition. Many studies provide new insights into the role of miRNAs and the pathogenesis of metastatic CC. In this review, we will offer an overview and update of our present understanding of the potential roles of miRNAs in metastatic CC.

## Introduction

Cervical cancer (CC) is one of the most commonly diagnosed cancers and is the third leading cause of cancer mortality amongst females [1]. An increasing amount of research has revealed that long-lasting infections of high-risk human papillomavirus (HPV), such as HPV-16 and HPV-18, mainly compose the majority of CC cases [2,3]. In fact, not all metastatic CC patients are diagnosed with HPV infection, which indicates that a number of indeterminate factors might contribute to the initiation and progression of CC [4–6]. Despite advances in surgery combined with radiotherapy and/or chemotherapy, some CC patients undergo early metastases of the primary tumor, especially lymph node metastasis (LNM), that lead to poor prognosis and poor therapeutic outcomes [7–10]. Hence, it is important to elucidate the molecular mechanisms underlying the metastasis of CC.

Based on sufficient research, numerous signaling pathways and molecules are involved in the metastasis of CC. For instance, the phosphatidylinositol 3-kinase/protein kinase-B (PI3K/AKT) signaling pathway, known as a key driver in carcinogenesis, plays an important role in the migration and invasion of CC [11,12]. Wnt/β-catenin, p38/MAPK, p53, and hedgehog signaling pathways were also reported to be related to carcinogenesis and progression in CC [13–17]. In addition, emerging molecules such as circular RNAs (circRNAs), long noncoding RNAs (lncRNAs), and exosomes were also shown to be related to the tumorigenesis of CC [18–20]. In recent decades, molecule-targetted therapy of CC has been developed. According to many studies, microRNAs (miRNAs) function to modulate the pathophysiologic mechanism in CC partly through the signaling pathways mentioned above, which might offer a new therapeutic method in the future and bring CC patients a hope for treatment [4,21,22].

miRNAs are a class of endogenous, highly conserved, noncoding RNAs (18–25 nts in length) that adjust gene expression both transcriptionally and post-transcriptionally [23,24]. They are involved in various physiological and pathological processes via binding to mRNAs at their 3′-UTRs [25,26]. Dysregulated miRNAs can be loosely divided into two groups: oncogenic miRNAs and oncosuppressor miRNAs. Both groups of miRNAs correlate with numerous biological processes such as invasion and metastasis in CC, thereby suggesting that miRNAs might serve as a set of novel biomarkers for the diagnosis and molecule-targetted therapy of metastatic CC.

Herein, we conclude that existing studies focus on the identification of miRNAs as diagnostic and prognostic markers for metastatic CC. Furthermore, we provide insight into the strategies for using miRNAs in metastatic CC therapy based on their putative functions.

## Dysregulated miRNAs in CC invasion and metastasis

A previous review summarized that miRNAs with altered expression patterns were related to oncogenic or tumor-suppressing functions in CC, and the differential miRNA expression pattern was closely contacted with complex CC progression [27]. In detail, the existence of oncogenic miRNAs or oncosuppressor miRNAs indicated that miRNAs played a promotive or suppressive role in the development of tumors. However, more detailed information about the role of miRNAs in the invasion and metastasis of CC is lacking. In the following section, we will further discuss the specific mechanism in which dysregulated miRNAs modulate the invasion and metastasis of CC through targetting genes.

## Oncogenic miRNAs in the metastasis of CC

Plentiful findings revealed that the autophagy-related protein (ATG) family plays crucial roles in autophagosome formation through communication between members of the ATG family [28,29]. For instance, ATG7 has been implicated in metastasis as one of the master regulators of the autophagy process and is responsible for autophagosome formation and vesicle progression [30]. Afterward, Zhao et al. [31] published that miR-20a functions as a promoter of metastasis via ATG7. Migration and invasion of CC were also found to be enhanced by miR-378 through ATG12 [32].

### Tissue inhibitors of metalloproteinases and matrix metalloproteinases

In 2017, Wei et al. [33] alleged that miR-21 participated in promoting LNM of CC, but they did not discuss the pathways of metastasis. In fact, during the process of invasion and metastasis, tissue inhibitors of metalloproteinases (TIMPs), particularly TIMP2 and TIMP3, were equipped to reverse the degradation of collagenous substrates in the surrounding extracellular matrix (ECM) by matrix metalloproteinases (MMPs) [34]. For example, miR-21 showed its ability to advance invasion of CC through suppressing TIMP3 [35]. In addition, miR-20a [31] as well as miR-106a [36] suppressed the migration and invasion of CC cells by targetting TIMP2.

### Other genes

**Figure 1:**
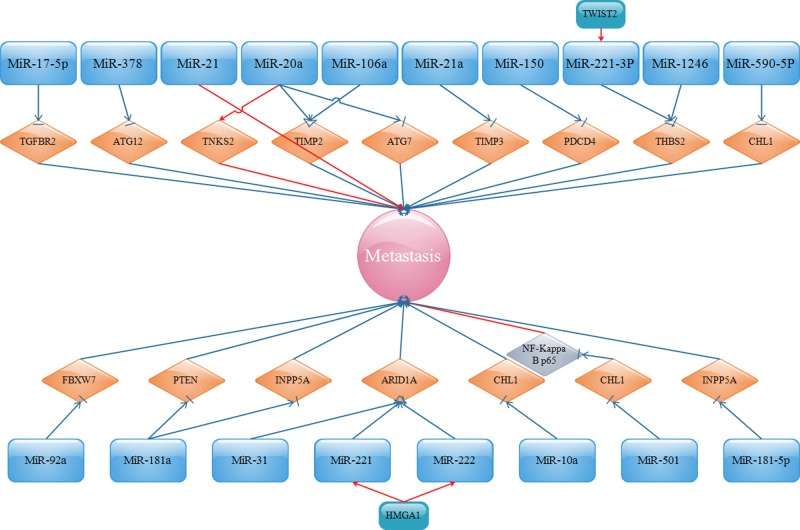
Oncogenic miRNAs and their targets in promoting metastasis

It was reported by Chen et al. [37] and Wei et al. [38] that miR-1246 and miR-221-3p facilitated the metastasis of CC via targetting thrombospondin-2 (THBS2, TSP2). THBS2 is a member of the thrombospondin family that regulates cell migration and inhibits tumor metastasis [39,40]. In addition, tankyrase 2 (TNKS2), which belongs to the human telomere-associated poly (ADP-ribose) polymerase (PARP) family, was claimed to increase telomere length, thus enhancing tumor progression [41,42]. miR-20a was announced to directly up-regulate TNKS2 and correspondingly strengthen the migration and invasion of CC [43]. Moreover, programmed cell death protein 4 (PDCD4), inhibited by miR-150, was attributed to the suppression of cancer cell migration and invasion [44]. miR-31 [45] and miR-221 [46], along with miR-222 [46], were verified as upstream of the AT-rich interactive domain-containing protein 1A (ARID1A), which is involved in the SWI/SNF family and recognized as a tumor suppressor in cancer through multiple kinds of pathways, such as p53 and PI3K/AKT pathways [47]. Furthermore, the positive effect of miR-221/222 on the metastasis of CC could also be exacerbated by high-mobility group AT-hook1 (HMGA1), an architectural transcription factor that directly binds to AT-rich regions in the minor groove of DNA4 [48]. Furthermore, miR-10a [49] and miR-590-5p [50] inversely correlated with the expression of a close homolog of l1 (CHL1), a putative tumor suppressor and a member of cell adhesion molecules (CAMs), that results in increased migration and invasion of CC. The migratory and invasive potentials of CC cells could also be activated by miR-501 by lowering the expression of cylindromatosis (CYLD) and subsequently stimulating NF-κB/p65 activation [51]. miR-92a functions as an onco-miRNA by targetting the F-box and WD repeat domain-containing 7 (FBXW7), thereby elevating the metastasis of CC [52]. The expression of miR-181a-5p was positively associated with the progression of CC through adversely targetting inositol polyphosphate-5-phosphatase A (INPP5A) [53]. In addition, the suppression of miR-181a, which was up-regulated in CC cell lines, evidently regulates metastasis of CC by regulating the PTEN/AKT/FOXO1 pathway [54]. MiR-19a/b were noted to be up-regulated in CC and promoted CC cell invasion through direct and negative regulation of Cullin-5 (CUL5) expression, termed as vasopressin-activated calcium mobilizing receptor (VACM-1) [55]. In general, according to the miRNAs mentioned above, detailed signaling pathways are shown in Figure 1.

## Oncosuppressor miRNAs in the metastasis of CC

### Mitogen-activated protein kinases

Mitogen-activated protein kinases (MAPKs) are crucial in modulating cancer cell invasion and metastasis and have been implicated in a wide range of physiological processes such as cell growth, differentiation, and apoptosis. HOX transcript antisense RNA (HOTAIR) attributes to the migration and invasion of CC via targetting the miR-23b/MAPK1 axis [56]. As a result, miR-23b plays an inhibitory role in the metastasis of CC. In addition, miR-329-3p [57] and miR-200c [58] suppress cell migration and invasion by targetting MAPK1 or MAPK4.

### MMPs

Additionally, the protective roles of miR-132 [59] and miR-183 [60] in the invasion of CC are related to the decreased production of MMP2 and/or MMP9. miR-454-3p restrained CC cell migration and invasion partly due to targetting c-Met, correspondingly leading to the down-regulation of p-AKT, MMP-2, and MMP-9, the downstream proteins of c-Met [61]. miR-486-3p is a significantly down-regulated miRNA in CC and functions to repress CC cell metastasis via down-regulating ECM1 [62]. The metastasis and invasion of CC can also be inhibited by miR-7 through targetting focal adhesion kinase (FAK), an important adhesion kinase that is related to ECM integrin signaling, cell motility, and proliferation [63].

### Vascular endothelial growth factor

Vascular endothelial growth factor (VEGF), a well-known tumor metastasis-driving factor, plays a crucial role not only in angiogenesis and vascular permeability but also in the function and trafficking of growth factor receptors and integrins [64]. The down-regulation of LncRNA UCA1 suspends VEGF expression with the introduction of miR-206 [65]. miR-144 exerts a suppressive impact on the migration and invasion of CC cells by targetting VEGFA and VEGFC, which belong to the VEGF family [66].

Up-regulated miR-375, miR-337, and miR-296 lessen CC cell malignant behaviors by targetting transcription factor specificity protein 1 (SP1), exerting many biological functions and contributing to the metastasis of CC [67–69]. SP1 is a member of the specificity protein/Krüppel-like factor (Sp/KLF) transcription factor family and plays a substantial role in the migration, invasion, and metastasis of various tumors.

MiR-424-5p, modulated by small nucleolar RNA host gene 12 (SNHG12), serves as a suppressor in metastatic CC [70]. In detail, a disintegrin and metalloproteinase 10 (ADAM10), an important mediator of cell signaling events, is commonly known as a contributor to the metastasis of oral squamous cell carcinoma (OSCC) [71] and osteosarcoma [72]. In fact, miR-140-5p plays a major part in the SNHG20-miR-140-5p-ADAM10-MEK/ERK axis, thus lessening the invasion of CC [73]. MiR-139-3p was recognized as a repressor of CC cell migration and invasion by reducing the expression of NIN1/RPNI2 binding protein 1 homolog (NOB1), a subunit of the 26S proteasome, and acting as an oncogene and inducing metastasis [74]. miR-138 significantly slows HeLa cell (a human CC cell line) migration by targetting human telomerase reverse transcriptase (hTERT), a catalytic subunit of telomerase involved in modulating telomerase activity [75]. Recently, Peng et al. [76] announced that brain cytoplasmic RNA 1 (BCYRN1), clearly up-regulated in CC, can increase invasion via adjusting the expression of miR-138 both *in vitro* and *in vivo*. It was demonstrated by Che et al. [77] that miR-107 also plays a suppressive role in the invasion of CC through targetting C–C chemokine receptor type 5 (CCR5), which is recognized as a mediator of chemotaxis and cellular homing and is involved in various biological processes such as the development of tumors. miR-107 also exhibits its inhibitory function in CC metastasis through another target, myeloid cell leukemia-1 (MCL1), an anti-apoptotic member of the Bcl-2 protein family and the activated ATR/Chk1 pathway [78]. Zhou et al. [79] found an adverse relationship between miR-145 levels and core transcription factors (TFs) such as Sox2, Nanog, and Oct4 and determined that high expression of miR-145 was able to inhibit invasion of tumor cells in CC. Thymic stromal lymphopoietin (TSLP), aberrantly highly expressed in CC cells, down-regulates the expression of miR-132 in CC cells and further induces the invasion of HeLa and SiHa cells, which are typical CC cells. The effects of miR-30a on the invasion of CC due to its inverse correlation with myocyte enhancer factor 2D (MEF2D), one member of the MEF2 family that is involved in the progression of various cancers [80]. ETS domain-containing protein Elk-1 (ELK1), which belongs to the ETS oncogene family and mediates transcriptional regulation, might rescue the inhibitory effects on migration and invasion activated by miR-326 [81]. Wang and Tian [82] also published that the overexpression of miR-206 inhibited B-cell lymphoma 2-associated athanogene 3 (BAG3), which is implicated in cell growth and metastasis and correspondingly reduces metastasis. miR-22 reduces CC cell invasion by targetting ATP citrate lyase (ACLY), which is effective in increasing metabolic capacity [83]. Overexpression of insulin-like growth factor 2 mRNA binding protein 1 (IGF2BP1) alters the suppressive role of both miR-124-3p [84] and miR-140-5p [85] on the malignant phenotypes of CC cells.

**Figure 2:**
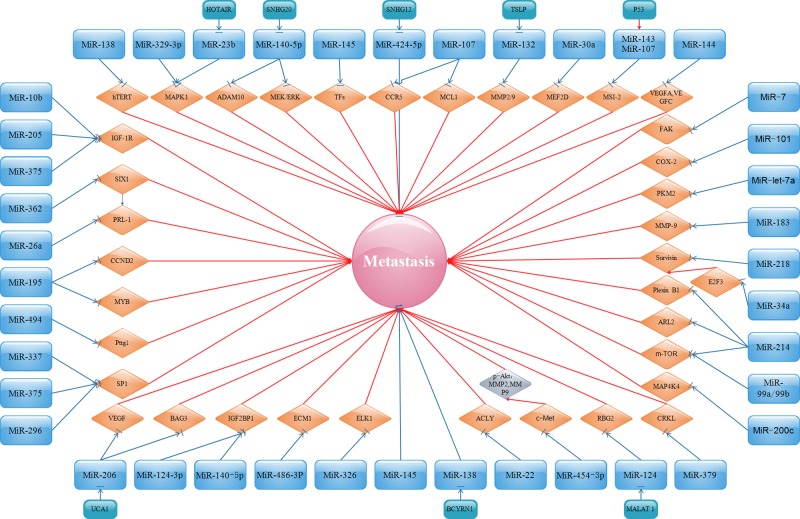
Oncosuppressor miRNAs and their targets in inhibiting metastasis

In addition, miR-124 is involved in the inhibition of CC cell invasion partly through the metastasis-associated lung adenocarcinoma transcript 1 (MALAT 1)-miR-124-RBG2 axis [86]. Insulin-like growth factor-1 receptor (IGF-1R), a transmembrane receptor that can enhance cell proliferation and differentiation through the PI3K/AKT and RAS/RAF/MEK/ERK signaling pathways, is involved in miR-10b-, miR-205-, or miR-375-mediated migration and invasion of CC cells [87–89]. The invasion of CC can be clearly suppressed by the up-regulation of miR-99a/b or miR-214 via modulating the mTOR signaling pathway [90,91]. Plexin-B1, as well as ADP ribosylation factors such as 2 (ARL2), negatively correlate with miR-214, and is shown to promote the invasion of HeLa cells [92,93]. The suppression of migration and invasion of CC, resulting from the overexpression of miR-362, was at least partly through the repression of the sineoculis homeobox homolog 1 (SIX1), a member of the homeodomain of the SIX families and related to development and progression of multiple tumors [94]. miR-494 suppresses CC invasiveness by targetting Pttg1, which is shown to induce a cell to enter the active cell cycle and promote cancer cell growth and metastasis [95]. Kogo et al. [96] and Geng et al. [97] declared that the miR-218/survivin or miR-34a/E2F3/survivin axis is pivotal in regulating migration and invasion of CC. E2F3 is a well-known transcription factor that regulates the cell cycle and cell differentiation and modulates the expression of survivin. MiR-let-7a inhibits CC cell migration and invasion via down-regulating pyruvate kinase muscle isozyme M2 (PKM2) [98]. The expression of phosphatase type IVA 1 (PRL-1) is inversely associated with miR-26a and can reverse the inhibitory effect of miR-26a on metastasis in CC [99]. miR-195 represses the expression of cyclin D2 (CCND2) and v-myb avian myeloblastosis viral oncogene homolog (MYB), thereby suppressing metastasis in CC [100]. CCND2 can regulate the cell cycle G_1_/S transition by communicating with cyclin-dependent kinases (CDKs). MYB is characterized as a cellular homolog of v-myb and a transforming oncogene in certain kinds of cancer. miR-101 negatively regulates cell migration and invasion in CC through inhibition of the target gene cyclooxygenase-2 (COX-2), which is positively involved in tumor development and progression [101,102]. miR-143/miR-107 elevated by p53 directly reduces the expression of Musashi-2 (MSI-2), resulting in the suppression of CC cell invasion [103]. miR-379 might act as a tumor suppressor in CC via negatively modulating V-crk avian sarcoma virus CT10 oncogene homolog-like (CRKL) [104]. miR-485, which is inversely associated with metastasis associated in colon cancer 1 (MACC1), was proven by Wang et al. [105] to inhibit the invasion of CC cells. Finally, based on the miRNAs mentioned above, related signaling pathways can be seen in Figure 2.

## The role of EMT-related miRNAs in the metastasis of CC

Epithelial–mesenchymal transition (EMT) is widely perceived as a phenotypic switch and allows the tumor to adopt a metastatic and invasive behavior with the down-regulation of E-cadherin and the up-regulation of N-cadherin, vimentin, and other EMT markers [106]. Many factors are extensively known to trigger the process of EMT, such as zinc finger E-box binding homeobox 1 (ZEB1), ZEB2, Snail1, and Snail2 [107,108]. According to Zaravinos [109], numerous signaling pathways play a substantial part in inducing or restraining EMT such as the TGF-β, Wnt, Hedgehog (Hh), Notch, integrin-linked kinase (ILK), and urokinase-type plasminogen activator receptor (uPAR) signaling pathways. Moreover, miRNAs such as miR-34a [110] and miR-200b [111] arise as regulators of EMT and thus modulate metastasis via certain signaling pathways. As discussed above, we have entered a new exciting era and have further explored the present research about the role of EMT-related miRNAs in the metastasis of CC.

### Oncogenic EMT-related miRNAs in the metastasis of CC

First, we discuss the promoting role of EMT-related miRNAs in the metastasis of CC.

TGF-β is known to play a complex and dichotomous role during tumorigenesis, functioning as a tumor suppressor in normal and early-stage cancers and as a tumor promoter in their late-stage counterparts. The switch in TGF-β function is known as the ‘TGF-β Paradox’, whose manifestations are linked to the initiation of EMT [112]. For instance, miR-17-5p advances the metastasis of CC cells by suppressing transforming growth factor-β receptor 2 (TGF-β R2), a member of the TGF-β signaling pathway [113]. Based on the same signaling pathway, miR-519d facilitates the metastasis of CC by down-regulating Smad7 [114], a member of the Smads family, is documented to play a pivotal role in co-ordinating tumor metastasis via the TGF-β/Smads signaling pathway [115].

**Figure 3:**
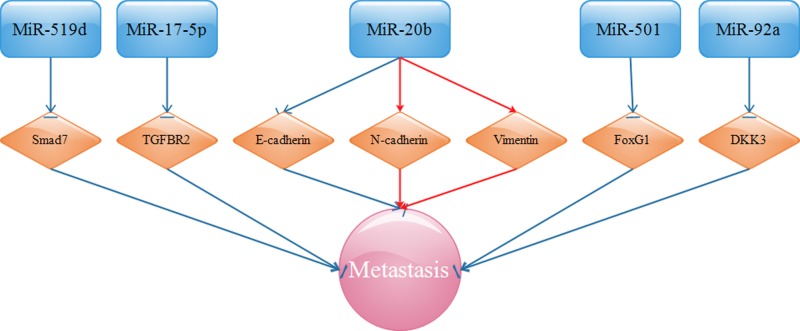
EMT-related miRNAs target TGF-β R2, Smad7, E-cadherin, N-cadherin, vimentin, Snail2, and FoxG1, and advance CC migration, invasion, and metastasis

Overexpressed miR-20b critically reinforces EMT by decreasing E-cadherin but increasing N-cadherin and vimentin and promoting metastasis [116]. The enrichment of miR-506, inversely modulated by circRNA 000284, prevents metastasis by inhibiting Snail2, an oncogene related to EMT [117]. The augmented expression of miR-92a was shown to eliminate the inhibitory effects of Dickkopf-related protein 3 (DKK3) on CC metastasis [118]. DDK3 acts as a vital tumor suppressor by interacting with the EMT-related Wnt signaling pathway and participating in many biological processes [119]. Up-regulated miR-200b is proposed to positively regulate the metastasis of CC via its definitely validated target, FoxG1 [120]. FoxG1 is perceived as a negative regulator of the TGF-β signaling pathway, thereby showing its oncogenic potential. Given the miRNAs mentioned above, we present these signaling pathways in Figure 3.

### Oncosuppressor EMT-related miRNAs in the metastasis of CC

We next explore the inhibitory role of EMT-related miRNAs in the metastasis of CC.

miR-142-3p functions in a tumor suppressive role in the invasion and EMT of CC cells through inhibition of the Frizzled 7 receptor (FZD7), vimentin, and Snail with up-regulation of E-cadherin [121]. FZD7, a member of Wnt receptors, is recognized as pivotal for the activation of both canonical and noncanonical Wnt pathways. Zhou et al. [122] noted that miR-212 plays its suppressive role in the metastasis of CC via inhibiting transcription factor 7-like 2 (TCF7 L2) expression. TCF7 L2 was affirmed as a new transcriptional factor and the critical factor in the Wnt signaling pathway, thus promoting EMT in tumor cells. miR-638 functions as a tumor suppressor in CC metastasis via modulation of the Wnt/β-catenin signaling pathway [123].

**Figure 4:**
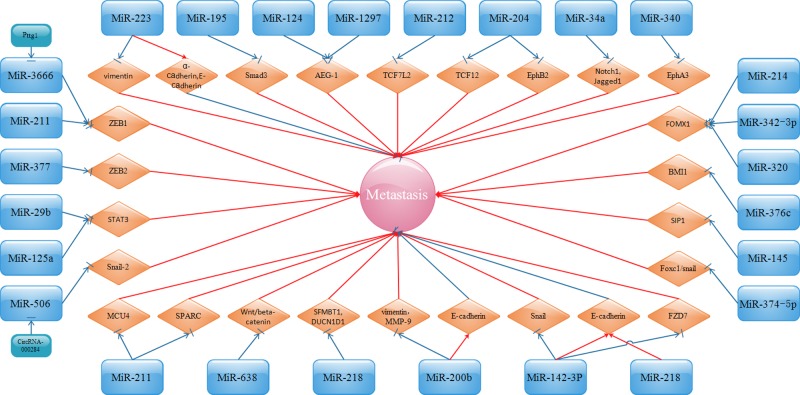
EMT-related miRNAs inhibit CC migration, invasion, and metastasis by targetting ZEB1/2, Smad3, AEG-1, TCF7 L2, MUC4, SPARC, Wnt/β-catenin, SIP1, SFMBT, Bcl-2, Bax, NF-κB, E-cadherin, α-cadherin, Notch1, Jagged1, TCF-12, EphB2, Vimentin, MMP-9, STAT3, FZD7, Snail, FOMX1, BMI1, and EphA3

The metastasis of CC was clearly inhibited by miR-3666 and miR-211 through the pituitary tumor-transforming gene 1 (*Pttg1*)/miR-3666/ZEB1 and the miR-211/ZEB1 [124,125] pathways, respectively. Additionally, miR-377 decreases the invasion of CC cells by inversely modulating the expression of ZEB2 [126]. miR-195, which is markedly down-regulated in CC and negatively correlates with Smad3, plays an apparently inhibitory role in CC cell migration and invasion [127]. Smad3 belongs to the Smad family and participates in TGF signaling. TGFβ/Smad3 is shown to induce EMT and the migration and invasion of CC cells. Research by Zhang et al. [128] showed that miR-124 is particularly down-regulated in CC and regarded as anti-miRNA and is involved in the inhibition of EMT and metastasis through directly targetting astrocyte-elevated gene-1 (AEG-1). AEG-1, also known as metadherin (MTDH), and lysine-rich CEACAM1 co-isolate protein (LYRIC) greatly participate in carcinogenesis and tumor progression in several malignancies. Fortunately, it was also discovered by Wang et al. [129] that alteration of AEG-1 eliminates the suppressive effects of miR-1297 on the metastasis of CC and EMT. The down-regulation of miR-145 might generate EMT and the metastasis of CC in conjunction with up-regulated expression of SMAD-interacting protein 1 (SIP1, also known as ZEB2) [130]. SIP1 is accepted as a strong suppressor of E-cadherin and is known to inhibit kinds of junctional complex genes, thus activating invasion and metastasis. miR-211 appears to be anti-miRNA due to its suppressive effect on Mucin 4 (MUC4) [131] and protein acidic and rich in cysteine (SPARC) [132], thus inhibiting CC cell invasion and reversing EMT properties. SPARC, which belongs to the matricellular family of secreted proteins, is related to cell matrix interactions and affects cell progression and might serve as an important factor in the EMT of CC. miR-218 inhibits EMT, migration, and invasion by targetting the 3′-UTRs of Scm-like with four MBT domains 1 (SFMFBT1) and defective in cullin neddylation 1 domain containing 1 (DCUN1D1) in CC [133]. Induced expression of miR-34a suppresses not only Notch1 and Jagged1 but also Notch signaling, thereby inhibiting the invasion capacity of CC cells [134]. miR-204 was verified by Shu et al. [135] as a metastasis-associated gene and might lead to the metastasis of CC via regulating transcription factor 12 (TCF12), a transcriptional repressor of E-cadherin. Moreover, miR-204 acts as a tumor suppressor in the metastasis of CC by directly targetting Ephrin type B receptor 2 (EphB2), which might promote the progression of tumors by inducing EMT and affecting its major downstream signaling pathway, PI3K/AKT [136]. The overexpression of miR-200b in CC cells decreases their migratory potential and EMT as shown by up-regulated E-cadherin and down-regulated vimentin and MMP-9 [137]. Li et al. [138] and Fan et al. [139] found that miR-29b, as well as miR-12 expression, participates in the inhibition of metastasis and EMT procedure of CC cells via targetting the signal transducer and activator of transcription 3 (STAT3) pathway, which performs a vital role in the cellular signaling pathway. The overexpression of Forkhead box M1 (FOXM1) can counteract the inhibitory influence of miR-214, miR-342-3p, and miR-320 on the metastasis of CC [140–142]. Highly expressed FOXM1 is positively associated with tumor metastasis and EMT. miR-374c-5p effectively inhibits the invasion and migration of CC cells and the process of EMT by targetting FOXC1, which belongs to the FOX transcription factor superfamily and greatly participates in EMT and tumor metastasis [143]. miR-376c affects CC metastasis by directly targetting B cell-specific Moloney murine leukemia virus insertion site 1 (BMI1), which might greatly affect EMT [144]. miR-340 is verified to slow the process of tumor metastasis by suppressing Ephrin receptor A3 (EphA3) [145,146]. This kind of regulation is dependent on the EMT pathway, since when miR-340 is overexpressed, the expression of E-cadherin increases and that of vimentin and α-SMA decreases. miR-223 up-regulates the epithelial markers E-cadherin and α-cadherin and down-regulates the mesenchymal marker vimentin, thus suppressing the metastasis of CC [147]. miR-218 overexpression inhibits cell migration partly due to the down-regulation of Bcl-2 and NF-κB and the up-regulation of Bax and E-cadherin [148]. Regarding the miRNAs mentioned above, associated signaling pathways are shown in Figure 4.

## The role of HPV-related miRNAs in the metastasis of CC

Persistent infection with HPV is acknowledged as one of the greatest risk factors for CC [149]. It is probable that the recent breakthroughs with respect to CC have come from the cognition that HPV silences tumor suppressor genes through HPV-encoded oncoproteins E6 and E7 (HPV E6 and HPV E7). Nonetheless, single HPV infection is not sufficient for the metastasis of CC, and some other HPV-related risk factors are emerging [150]. A number of studies have confirmed that the expression of miRNAs is closely related to HPV, mainly through HPV E6 and HPV E7 [151].

Given that multiple oncosuppressor miRNAs such as miR-99a/b [90], miR-214 [91], and miR-21 [152] are suggested to communicate with the mTOR pathway, we further discuss the interaction between HPV and mTOR. It is recognized that mTOR plays pleiotropic pathogenetic roles not only in different types of cancer including breast cancer [153] and in the development of chemoresistance [154] but also in autoimmune diseases [155,156] and viral diseases such as HIV [157,158]. In 2010, Spangle and Munger [159] showed that HPV16 E6-mediated activation of mTORC1 signaling might result in the promotion of protein synthesis. In fact, as early as 2012, mTOR has become a potential therapeutic target in HPV-associated oral and cervical squamous carcinomas [160]. In addition, mTOR downstream effectors 4EBP1 and eIF4E, which control protein synthesis initiation, are closely correlated with oncogenic HPV types [161]. In an inducible HPV-16 E6/E7 mouse model, mTOR inhibition via rapamycin protected HPV-E6/E7-expressing tissues from carcinogen-induced malignancies [162].

Next, we discuss the role of HPV-related miRNAs in the metastasis of CC. miR-27b, up-regulated by HPV E7, functions to inhibit the expression of peroxisome proliferator-activated receptor γ (PPARγ), a tumor suppressor [163], and to promote invasion of CC cells [164]. miR-20b, up-regulated by HPV E6, acts to restrain TIMP2, thus advancing invasion of CC cells [116]. In addition, HPV E6 promotes CC metastasis by modulating miR-218, thus targetting SFMFBT1 and DCUN1D1 [133]. SFMBT1, a member of the malignant brain tumor (MBT) domain-containing protein family, participates in multiple cellular processes including cell metastasis. DCUN1D1 is recognized as an oncogene and is overexpressed in many types of malignant tumors that leads to a series of diseases including cancers.

**Figure 5:**
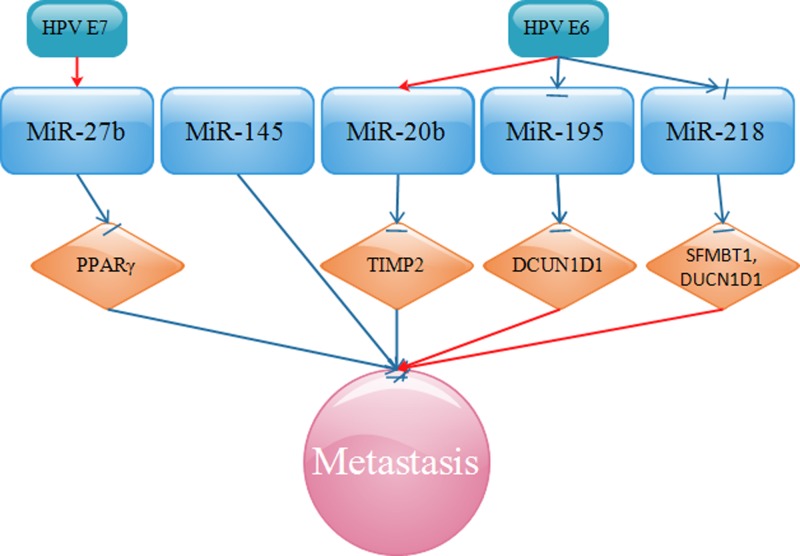
HPV-related miRNAs regulate CC migration, invasion, and metastasis by targetting PPARγ, TIMP2, DCUN1D1, and SFMBT1

In contrast with the miRNAs mentioned above, we next summarized miRNAs that are inversely associated with metastasis of CC. Shi et al. [165] published that a novel HPV-E6-p53-miR-145 pathway plays an important part in the modulation of CC cell invasion. miR-195, targetted by oncogenic HPV E6, negatively mediates CC cell migration and invasion partly through defects in cullin neddylation 1 domain containing 1 (DCUN1D1), which is significantly up-regulated in CC [166]. All the miRNAs related to HPV in the metastasis of CC are shown in Figure 5.

### The role of miRNAs *in vivo* and the diagnosis and treatment of metastatic CC

To date, many studies have been carried out to verify whether miRNAs could play biological functions in *in vivo* models of CC. Luckily, it was verified that induced expression of miR-let-7a [98], miR-17-5p [113], miR-26a [99], miR-138 [75], miR-145 [79], and miR-206 [82] indeed inhibit the growth of *in vivo* tumor xenografts of CC. Furthermore, both miR-22 [83] and miR-140-5p [85] significantly suppress not only tumor growth but also metastasis in nude mice. However, silencing miR-200b notably inhibits *in vivo* tumor growth of CC [120]. In addition, overexpressed miR-21 results in an increase not only in the size of tumors but also in the frequency of lymph node metastasis [33].

With regard to the diagnosis and treatment of metastatic CC, researchers have studied cervical tissues and found a relationship between miRNAs and the diagnosis and treatment of metastatic CC. It was of interest to find that decreased miR-99a/b [90], miR-125a [139], miR-138 [75], miR-140-5p [85], miR-144 [66], miR-195 [127], miR-205 [88], miR-214 [91], miR-218 [96,133,148,167], miR-329-3p [57], miR-337 [68], miR-362 [94], miR-374c-5p [143], miR-375 [67], miR-377 [126], miR-379 [104], miR-485 [105], miR-486-3p [62], miR-638 [123], and miR-1297 [129] expression strongly correlate with tumor size, TNM stage, tissue pathology grade, International Federation of Gynecology and Obstetrics (FIGO) stage, lymph node metastasis, or distant metastasis in patients with CC. In addition, overexpressed miR-20a [31], miR-21 [168], miR-92a [118], miR-145 [79], miR-195 [166], miR-199b-5p [169], and miR-501 [51] closely correlate with histological grade, tumor diameter, overall survival (OS), progression-free survival (PFS), late FIGO stages, lymph node metastasis, or preoperative metastasis. Based on the above discussion, we considered that miRNAs might function as effective tools or potential markers with utility in advances in the diagnosis and treatment of metastatic CC.

## Conclusion

miRNA-based cancer therapy is a relatively new concept, and emerging studies are starting to show the potential roles of miRNAs in the possible clinical therapy for human malignancies. miRNAs have been found to play an important role in the metastasis of cancers such as breast cancer [170,171]. Accompanied with the above studies, a preliminary understanding demonstrates the intrinsic features and biological functions of miRNAs during the metastasis of CC. From Figures 1 to 5, it is easy for us to distinguish miRNAs between those communicating with oncogenes or tumor suppressor genes and those affecting invasion and metastasis. miRNAs have a vital role in all stages of CC progression from cell invasion and migration to eventual tumor metastasis. Because miRNAs are comprehensively associated with the metastasis of CC, intensive research on the roles of miRNAs is urgently needed, which will provide novel probable targets for the development of therapies for CC.

In recent years, the rapid development of miRNA profiling microarray chips and high-throughput sequencing have shown a great advantage in accelerating the study of the relationship between CC and miRNAs. Secreted miRNAs in serum could be detected for cancer diagnosis, including early metastasis of CC based on alterations in various miRNA serum levels. Furthermore, according to advances in the depth of sequencing and the recognition of tumor metastasis, miRNAs interact with other molecules previously unknown to us such as extracellular vesicles (EVs), circRNAs, and lncRNAs. These molecules, along with miRNAs, have been found to function together to modulate the progression of cancers [172–174].

Thus, miRNA-based therapy may be possible, as there are many approaches to miRNA-specific personalized treatment and molecular targetted therapy. In the meantime, it might be a potential future anticancer therapy by regulating the expression of oncogenic miRNAs.

## Abbreviations

3′-UTR: 3′-Untranslated region
AEG-1: Astrocyte-elevated gene-1
ACLY: ATP citrate lyase
ADAM10: A disintegrin and metalloproteinase 10
ARF: ADP-ribosylation factor
ARID1A: AT-rich interactive domain-containing protein 1A
ARL2: ADP-ribosylation factor like 2
ATG: Autophagy-related protein
ATR/Chk1: ATM- and RAD2-related/Chk1
BAG3: B-cell lymphoma 2-associated athanogene 3
Bcl-2: B-cell lymphoma-2
BCYRN1: Brain cytoplasmic RNA 1
BIRC5: Survivin
BMI1: B-cell-specific moloney murine leukemia virus insertion site 1
CAM: Cell adhesion molecule
CC: cervical cancer
CircRNA: Circular RNA
CCND2: Cyclin D2
CCR5: C–C chemokine receptor type 5
CDK: Cyclin-dependent kinase
CHL1: Close homolog of l1
circRNA: Circular RNA
CYLD: Cylindromatosis
COX-2: Cyclooxygenase-2
CRKL: V-crk avian sarcoma virus CT10 oncogene homolog-like
CUL5: Cullin-5
DCUN1D1: Defective in cullin neddylation 1, domain containing 1
DDK3: recombinant human dickkopf-related protein 3
DKK3: Dickkopf-related protein 3
E2F3: E2F transcription factor 3
ECM: Extracellular matrix
eIF4E: eukaryotic translation initiation factor 4E
ELK1: ETS domain-containing protein Elk-1
EMT: Epithelial–mesenchymal transition
EphA3: Ephrin receptor A3
EphB2: Ephrin type B receptor 2
EphA3: Ephrin receptor A3
EV: Extracellular vesicle
FAK: Focal adhesion kinase
FBXW7: F-box and WD repeat domain-containing 7
FIGO: International Federation of Gynecology and Obstetrics
FoxG1: forkhead box G1
FOXM1: Forkhead box M1
FZD7: Frizzled7 receptor
Hh: Hedgehog
HMGA1: High-mobility group AT-hook1
HOTAIR: HOX transcript antisense RNA
HOX: homeobox
HPV: Human papillomavirus
hTERT: ATCC human telomerase reverse transcriptase
hTERT: Human telomerase reverse transcriptase
IGF2BP1: Insulin-like growth factor 2 mRNA binding protein 1
IGF-1R: Insulin-like growth factor-1 receptor
ILK: Integrin-linked kinase
INPP5A: Inositol polyphosphate-5-phosphatase A
LNM: Lymph node metastasis
MACC1: Metastasis associated in colon cancer1
MALAT 1: Metastasis-associated lung adenocarcinoma transcript 1
MAPK: Mitogen-activated protein kinase
MAP4K4: Mitogen-activated protein kinase kinase kinase kinase 4
MBT: Malignant brain tumor
MCL1: Myeloid cell leukemia-1
MEF2D: Myocyte enhancer factor 2D
MiRNA: MicroRNA
MMP: Matrix metalloproteinase
mRNA: Messenger RNA
MSI-2: Musashi-2,
mTOR: Rapamycin
mTOR: mechanistic target of rapamycin
mTORC1: mammalian target of rapamycin complex-1
MUC 4: Mucin 4
MYB: V-myb avian myeloblastosis viral oncogene homolog
NF-κB: nuclear factor-kappa B
NOB1: NIN1/RPNI2 binding protein 1 homolog
OS: Overall survival
PARP: Human telomere-associated poly (ADP-ribose) polymerase
PDCD4: Programmed cell death protein 4
PEBP1: phosphatidylethanolamine binding protein 1
PFS: Progression-free survival
PI3K/AKT: Phophatidylinositol 3-kinase/protein kinase-B
PKM2: Pyruvate kinase muscle isozyme M2
PPARγ: Peroxisome proliferators-activated receptor γ
PRL-1: Phosphatase type IVA 1
PTEN/FOXO1: phosphatase and tensin homolog deleted on chromosome ten/forkhead box O1
Pttg1: Pituitary tumor-transforming gene 1
SCC: Squamous cell carcinoma
SFMBT1: Scm-like with four MBT domains 1
SIP1: SMAD-interacting protein 1
SIX1: Sineoculis homeobox homolog 1
SNHG12: Small nucleolar RNA host gene 12
SPARC: Protein acidic and rich in cysteine
Sp1: Specificity protein 1
STAT3: Signal transducer and activator of transcription 3
SWI/SNF: switch in mating type/sucrose non fermentation
TCF7L2: Transcription factor 7-like 2
TCF12: Transcription factor 12
TF: Transcription factor
TGFβR2: Transforming growth factor-β receptor 2
THBS2, TSP2: Thrombospondin-2
TIMP: Tissue inhibitor of metalloproteinase
TNKS2: Tankyrase 2
TSLP: Thymic stromal lymphopoietin
TWIST2: Twist homolog 2
UCA1: Urothelial cancer associated 1
VACM-1: Vasopressin-activated calcium mobilizing receptor
VEGF: Vascular endothelial growth factor
VEGFA: Vascular endothelial growth factor A
ZEB1: Zinc finger E-box binding homeobox 1
ZEB2: Zinc finger E-box-binding homeobox 2
