# Expression of Concern: The role of miRNAs in the invasion and metastasis of cervical cancer

**DOI:** 10.1042/BSR20181377_EOC

**Published:** 2026-11-04

**Authors:** Jin-yan Wang, Li-juan Chen

**Affiliations:** 1 Department of Obstetrics and Gynecology, Zhangjiagang First People's Hospital, Zhangjiagang 215600, Jiangsu, P.R. China; 2 Department of Oncology, The First Affiliated Hospital of Nanjing Medical University, Nanjing 210029, P.R. China

**Keywords:** cervical cancer, invasion, metastasis, MicroRNAs

*Bioscience Reports* has been made aware by a reader of potential issues surrounding the scientific validity of some of the references cited within this paper (DOI: 10.1042/BSR20181377) and is hence issuing a permanent Expression of Concern to notify readers.

Seventeen articles cited have been retracted since the publication of this review, and one retracted prior to the review's publication. These include the following:

- DOI: 10.3892/mmr.2022.12888
- DOI: 10.3892/ijmm.2021.4973
- DOI: 10.3892/etm.2018.5714
- DOI: 10.26355/eurrev_201804_14711
- DOI: 10.1042/bsr20171563
- DOI: 10.3727/096504017X15132494420120
- DOI: 10.3892/or.2024.8745
- DOI: 10.3892/or.2017.5979
- DOI: 10.3892/ol.2023.14168
- DOI: 10.1002/jcb.26414
- DOI: 10.3892/etm.2017.5528
- DOI: 10.1038/s41419-017-0077-5
- DOI: 10.3892/or.2024.8713
- DOI: 10.1007/s13277-015-3440-0
- DOI: 10.1016/j.canlet.2021.07.003
- DOI: 10.1177/1010428317711323
- DOI: 10.32604/or.2024.056126
- DOI: 10.1038/s41419-026-08531-5

The authors were contacted regarding the concerns but did not respond. As such, the article will remain published but with an associated Expression of Concern.

